# Mrs Gillick and the Wisbech Area Health Authority

**Published:** 1985-04

**Authors:** John Griffiths


### Mrs Gillick and the Wisbech Area Health Authority

**John Griffiths,** Solicitor

In December 1984 Mrs Victoria Gillick was the mother of five girls under the age of 16. Four years previously the Department of Health and Social Security in a circular to area health authorities had advised that family planning clinic sessions should be available to everyone irrespective of age: that attempts should be made to persuade children under the age of 16 attending such clinics to involve their parents or guardians: and that although it would be most unusual to provide such children with contraceptive advice and treatment without parental consent, in exceptional cases it was for the doctor alone, in the exercise of his clinical judgment, to decide whether contraceptive advice or treatment should be given to them. Mrs. Gillick sought from the High Court a declaration that the advice given in the circular was unlawful: she lost her case. She went to the Court of Appeal whose three judges were unanimous in reversing that decision, but who gave the area health authority leave to appeal to the House of Lords where the matter may be finally canvassed: what follows therefore examines the law as it now stands.

The general principle, derived from the English common law, is that the parent or parents or guardian or guardians of a child have the duty of care and the right of custody and control of that child from birth to majority: until 1969 majority was attained at the age of 21: the age of 18 was substituted by the Family Law Reform Act 1969, so that now for all purposes and in all respects the parental duty of care and right of custody and control ceases at the latter age. Numerous qualifications of this general principle have always existed, some created by statute and others by the common law itself: that is the evolving law which is made by the judges by reference to public policy, custom, precedent, natural justice, contemporary opinion and a host of other considerations but always subject to Acts of Parliament. The exceptions directly relevant to the issue litigated by Mrs. Gillick are first, a provision of the Family Law reform Act 1969 that a minor aged 16 or over may validly consent to surgical, medical or dental treatment: and second, that at common law a child who had attained 'the age of discretion' might give a valid consent to certain processes hitherto confined to the custody of the child. Apart from the statutory and common law exceptions the parental authority is absolute: it lasts until the minor attains the age of 18: and may be interfered with only by the Court. It could not be doubted, said the Court of Appeal, that up to some age no one save the Court is entitled to interfere with parental rights: and the only question to be determined is what that age is. As a result of the express statutory provision a minor can give a valid consent to surgical, medical or dental treatment from the age of 16 onwards: but this provision does not, except perhaps by implication, exclude the validity of a consent given before that age. Logically, therefore, the possible answers to the question are (a) some age between birth and 'the age of discretion'; (b) the 'age of discretion' and (c) the age of 16. There is neither statutory nor common law authority supporting the suggestion that any interference otherwise than by the Court with parental rights over a child between birth and the 'age of discretion' is permissible: so that the first of the possible answers to the question must be rejected. So far as the second possible answer is concerned, the 'age of discretion' is a phrase descriptive rather than definitive: and the authorities indicate that its invocation to validate a choice made by a child has hitherto been limited to cases involving custody: so that if on the grounds of vagueness and its limited application hitherto the second answer be excluded, the third answer seems to be the correct one.

The Court of Appeal, however, did not rely only upon some such process of elimination to reach its conclusion. The Court referred, to aid it in ascertaining public policy with a regard to children in the context of sexual matters, to the criminal law. Since 1885 sexual intercourse with a girl under the age of 16 has been a crime, and the consent of the girl has not afforded a defence: the Court said that it would be wholly incongruous, when the act of intercourse is criminal and when (as is the case) permitting it to take place on one's premises is criminal, that the area health authority should provide facilities which would enable girls under the age of 16 the more readily to be parties to the commission of such acts: and concluded that as a matter of common law a girl under the age of 16 can give no valid consent to anything which would constitute an assault, such as an examination or the fitting of a contraceptive device: nor to the giving of advice on contraception or abortion or the prescription of contraceptive drugs, such advice or prescription being an interference with the parental rights. The proper course for the adviser said the Court of Appeal, is either to seek the content of the parents irrespective of the wishes of the child, or to apply to the Court. Almost as an afterthought, the Court qualified this statement of the law by pronouncing that none of it would apply 'in emergency'.

In a few months' time, this decision may be reversed by the House of Lords; the evolving common law may be viewed differently by that tribunal. The express provision of the Family Law Reform Act 1969 that a girl of 16 or over may validly consent to medical, surgical or dental treatment does not necessarily exclude the possibility that if she has attained the 'age of discretion', whatever that age may be, she can give valid consent at common law to such treatment: and it is arguable that advice, as distinct from the administration of drugs or physical examination or operation, is not 'treatment' within the Act, and may be given to a girl under the age of 16 who has attained the 'age of discretion' without parental consent. There are cogent arguments for the proposition that the evils resulting from the decision are far greater than those arising from the exclusion of the parents from the doctor and patient relationship in this particular context: that one of the objects of the criminal law since 1885 is to prevent unwanted pregnancies of girls under 16, now far more effectively achieved by contraception: and that the object of the advice in the circular is identical with that of the criminal law in 1885. The House of Lords taking into account contemporary public policy, opinion and sexual attitudes may come to the conclusion that the common law has been wrongly stated by the Court of Appeal.

The decision poses a number of problems: what is an 'emergency': for instance, would an unwanted pregnancy continued by a girl of 15 to within a few days of the expiry of the time during which an abortion might lawfully be performed, justify a doctor in performing it without the consent of the girl's parents or the Court? Who is to ajudicate on the question: the doctor or the Court, necessarily after the doctor has made his decision, and given the advice or treatment? In the unlikely event of the doctor deciding to apply to the Court will the area health authority pay his costs? What should be the doctor's response to the parent of a 15 year old girl who brings the girl to him for contraceptive treatment which the girl refuses to receive: if he forces the treatment on her, is he guilty of an assault: if he refuses to provide the treatment is he liable to the parent or otherwise for the consequences? Lastly, how strict should a doctor be in verifying the girl's age: may he accept her own statement or should he insist upon independent evidence?

It is impossible to answer these questions with confidence but the solutions now offered seem to at least to conform with common prudence and common sense even if they be found ultimately to offend the common law.

Prudence requires that unless and until the decision of the Court of Appeal is reversed or modified by the House of Lords or by statute, the medical profession should act strictly in accordance with it, at the risk otherwise of civil litigation or criminal prosecution by the authorities, or what is more likely, at the instance of a pressure group. The doctor must assume that he alone is the judge of what constitutes an 'emergency' with the risk that his judgment may, whichever way it goes, be called in question after the event in civil or criminal proceedings. The doctor would be most unwise to place the slightest reliance upon his receiving from the area health authority or anyone else his costs of any application which he might make to the Court for assistance: or upon his receiving such assistance in time for it to be of any value. The doctor would take a very serious risk were he to advise or treat a girl of the 'age of discretion' but under the age of 16 at the behest of a parent but against the girl's will: his refusal entailing some risk perhaps of litigation at the instance of the parent or even the girl herself at a later date. So far as proof of the girl's age is concerned there is a tendency for the law to exclude as an available defence a belief, even when induced by the child, that he or she has attained a particular age: and in the present state of the law a doctor who is confronted with a doubtful case would be well advised to insist upon the production of a birth certificate and also perhaps some evidence that his patient is the girl to whom the certificate refers.

Accustomed as it is to the daily acceptance of onerous responsibilities and exposure to ill informed and sometimes prejudiced criticism the medical profession would be justified in demanding further legislation on this topic: but who would be prepared to assert that the result would not be confusion worse confounded?

Mr. Griffths will be glad to deal through our columns with any correspondence which may result.

